# Leiomyosarcoma as a Second Metachronous Malignant Neoplasm Following Colon Adenocarcinoma. A Case Report and Review of the Literature

**DOI:** 10.1080/13577140120048926

**Published:** 2001-03

**Authors:** A. Mavrodontidis, Ch. Zalavras, A. Skopelitou, V. Karavasilis, E. Briasoulis

**Affiliations:** 1 Department of Orthopaedic Surgery Ioannina University Hospital Ioannina Greece; 2 Department of Pathology Ioannina University Hospital Ioannina Greece; 3 Department of Medical Oncology Ioannina University Hospital Ioannina Greece; 4 Department of Medical Oncology School of Medicine University of Ioannina Kleisouras 4 Ioannina 453 33 Greece

## Abstract

Long-term cancer survivors are at increased risk for the development of second primary malignancies. This is usually associated with common genetic and etiologic factors and the treatment modality used for the primary cancer. In this paper we describe the case of a patient who developed a leiomyosarcoma in his left arm 5 years after he had a colon adenocarcinoma resected. Both primary tumours were treated successfully with surgical resection alone. The literature regarding second primary neoplasms, specifically focused on sarcomas, is briefly reviewed.


*Sarcoma* (2001) **5**, 31–33

CASE REPORT

### Leiomyosarcoma as a second metachronous malignant neoplasm following colon adenocarcinoma. A case report and review of the literature

A. MAVRODONTIDIS,[1] CH. ZALAVRAS,[1] A. SKOPELITOU,[2] V. KARAVASILIS[3] & E. BRIASOULIS[3]

[1]*Department of Orthopaedic Surgery,* [2]*Department of Pathology, and* [3]*Department of Medical Oncology, Ioannina University Hospital, Ioannina, Greece*

**Abstract**

Long-term cancer survivors are at increased risk for the development of second primary malignancies. This is usually associated with common genetic and etiologic factors and the treatment modality used for the primary cancer. In this paper we describe the case of a patient who developed a leiomyosarcoma in his left arm 5 years after he had a colon adenocarcinoma resected. Both primary tumours were treated successfully with surgical resection alone. The literature regarding second primary neoplasms, specifically focused on sarcomas, is briefly reviewed.

**Key words:** *leiomyosarcoma, second-primary, colon-adenocarcinoma, second-malignancy*

### Introduction

Second primary malignancies can develop after successful treatment of malignant neoplasms.[1] Occurrence of second malignancies has been associated with common etiologic factors implicated in the development of a first cancer, or with the treatment modality used for the primary cancer.[2,3] However, in many cases, such a relationship fails to be documented.[4] We present in this paper the case of a leiomyosarcoma that occurred 5 years following the resection a colon cancer. To our knowledge, this is the first report of such an occurrence.

### Case report

A 70-year-old male had a right hemicolectomy for a colon adenocarcinoma at the age of 65. Histological examination revealed a colon adenocarcinoma penetrating to the subserosa (Dukes' stage B) arising from a villous adenoma. The patient was not offered adjuvant chemotherapy but was only put on a regular follow-up consisting of clinical examination and laboratory evaluation every 6 months and colonoscopy every 2 years. Five years later, he developed a lump, gradually increasing in size, in his left arm. The swelling was located at the posterolateral surface of the upper third of his left arm and measured approximately 5 x 6 cm2 when he was seen in clinics 4 months later (Fig. 1). The mass was hard, firmly attached to the surrounding tissues and mildly painful at palpation, and the overlying skin was warm and

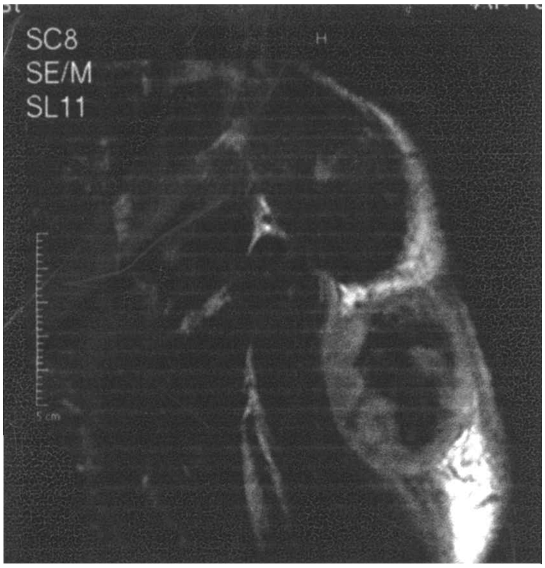

**Fig. 1.** *A magnetic resonance T1-weighted image showing a well-defined solid tumour with slightly hyperintense periphery and hypointense centre, in contact with the deltoid and the triceps muscles, that was histologically proven to be a grade III leimy osarcoma.*

Correspondence to: Evangelos Briasoulis, MD, Department of Medical Oncology, School of Medicine, University of Ioannina, Kleisouras 4, 453 33 Ioannina, Greece. Tel: (+30) 651-74973; Fax: (+30) 651-73368; E-mail: ebriasou@otenet.gr

1357–714X print/1369–1643 online/01/010031–03 © 2001 Taylor & Francis Ltd
DOI: 10.1080/13577140120048926

erythematous. The patient had a temperature of 37.5µC. Dynamic contrast-enhanced perfusion magnetic resonance imaging disclosed a well-defined solid tumour measuring 5 x 5.5 x 4.5 cm3, slightly hyperintense in the periphery with hypointense patches in the centre on T1-weighted images (Fig. 1), and highly hyperintense on T2-weighted images. The tumour was located by the deltoid muscle and in contact with the triceps, and with indications of possible infiltration of the subcutaneous fat and the long and lateral heads of the triceps muscle. Staging computed tomography scans of thorax and abdomen was proven negative for metastases. The tumour was widely excised together with the surrounding soft tissues and overlying skin, and with part of the deltoid and of the long and lateral heads of the triceps. A split thickness skin graft was used to cover the resulting skin defect. Microscopic examination showed a spindle cell sarcoma composed of short fascicles of cells, most of which had perinuclear vacuoles and/or clotted appearance of cytoplasm (Fig 2). Mitotic figures exceeded a number of 50 per 10 high power fields (x 40) and the extent of necrosis reached 30% of the tumor volume. Immunohistochemistry performed on paraffin sections detected strong expression of vimentin, muscle-specific actin (HHF35) in most neoplastic cells, while desmin was only focally expressed. EMA, Cytokeratins, S100 protein, and CD34 were all negative. Based on the morphological and immunohistochemical findings, the diagnosis of a grade III leiomyosarcoma was given. Resection margins were tumour free at a minimum of > 2 cm. No adjuvant therapy was administered and the patient remains disease-free 3 years after the sarcoma resection. One year following the sarcoma diagnosis, his brother died of oesophageal carcinoma at the age of 67.

### Discussion

Long-term cancer survivors are at increased risk for the development of second malignancies. The occurrence of a second metachronous primary malignancy may reflect an underlying genetic or immunological defect in the patient, environmental exposure to carcinogens or a complication of treatment modalities used in the management of the primary tumour.[1]

Radiotherapy has been associated with increased incidence of second malignancies, especially sarcomas. Post-irradiation sarcomas arise within areas exposed to prior irradiation. Of these, malignant fibrous histiocytoma is asas the most common histological type in adults,[5,6] while paediatric patients who received more than 6000 rad to the bone in childhood have a 40-fold increased risk for bone cancer.[7]

Chemotherapy-induced malignancies have been consistently reported in patients treated with alkylating agents in the past. Large increases in the incidence of leukaemia and non-Hodgkin's lymphomas, during the early years after treatment, and of solid tumours in the long-term have particularly been demonstrated in patients with Hodgkin's disease.[8] Smoking is another well known common factor to the development of a second primary in cancer survivors, with an overall cumulative risk of a second primary lung cancer of 4.7% and any tobacco-related tumor

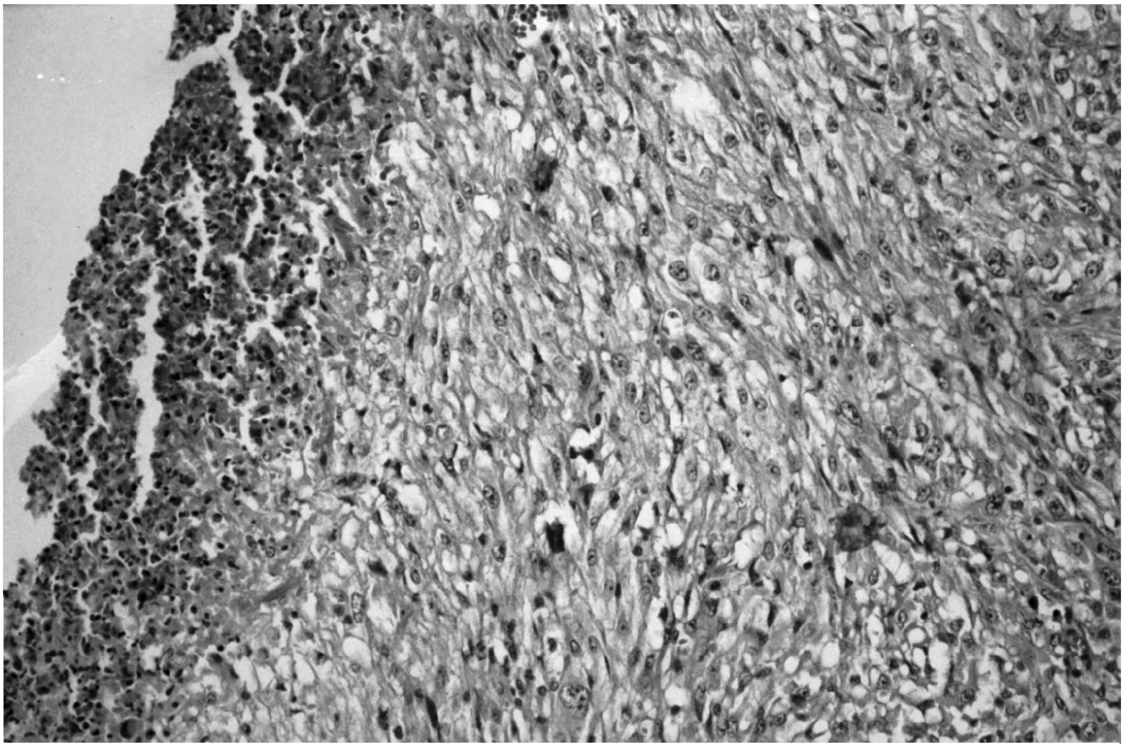

**Fig. 2.** *Photomicrograph of a tumour section adjacent to central necrosis. Spindle-shaped neoplastic cells are firmly packed and most of them have clear cytoplasm (H & E, x 300)*

of 11% at 10 years.[9] Second primary malignancies may also occur in the absence of recognizable etiologic or triggering factors. A 5% incidence of second primary neoplasms other than melanoma was found at 5 years in malignant melanoma survivors who received only surgical treatment,[10] while a moderately increased risk for the developing of meningioma was found in colorectal cancer surviving patients.[11] Genetic susceptibility for the development of non-treatment-related metachronous second primary malignancies is well known in definite tumour types such as retinoblastoma and Wilm's tumour, and is strongly suggested in patients with a family history of malignant neoplasms, especially at young ages. A small group of these patients has been shown to carry germ line mutations to the p53 gene.[12,13]

Second primary sarcomas are most often unrelated to irradiation, although they are commonly perceived as a late sequel of irradiation. Robinson *et al.* presented the clinical characteristics of 240 patients with sarcoma as a second metachronous primary neoplasm registered during the period 1973–1986 in the Surveillance, Epidemiology and End Results (SEER) Program in the United States. Only 30% of this group of patients had a post-irradiation sarcoma, while the others developed the sarcoma in an area not previously exposed to radiotherapy. In the SEER Program, the survival of patients with sarcoma as a second tumour was significantly worse in comparison with single sarcomas.[14] Sarcomas constitute the most common type of second malignant neoplasms in patients with a history of bilateral retinoblastoma, while bone tumours are ranked high in rate among second primary malignancies in patients with a history of Hodgkin's disease.[8,15] Furthermore, an increased incidence of Classic Kaposi's sarcoma was detected following diagnosis of lymphomas and breast cancer,[16] and of post-irradiation soft tissue sarcomas in patients with a history of testicular cancer.[17]

We conclude that, besides being the first report of a soft tissue sarcoma that occurs as a second metachronous neoplasm, following colorectal carcinoma, this case illustrates the worthiness of increased awareness for early diagnosis of a second primary malignancy in long-lasting disease-free survivors. Efforts for early detection and surgical treatment of secondary primary neoplasms may further improve prognosis in cancer patients.

### References

1 Meadows AT, Baum E, Fossati-Bellani F, *et al.* Second malignant neoplasms in children: an update from the Late Effects Study Group. *J Clin Oncol* 1985; 3(4):532–8.

2 de Kony SJ, Vathaire F, Chompret A, *et al.* Radiation and genetic factors in the risk of second malignant neoplasms after a first cancer in childhood. *Lancet* 1997; 350:91–5.

3 Kingston JE, Hawkins MM, Draper G J, *et al.* Patterns of multiple primary tumours in patients treated for cancer during childhood. *Br J Cancer* 1987; 56(3):331–8.

4 Shah S, Evans DG, Blair V, *et al.* Assessment of relative risk of second primary tumors after ovarian cancer and of the usefulness of double primary cases as a source of material for genetic studies with a cancer registry. *Cancer* 1993; 72(3):819–27.

5 Robinson E, Neugut AI, Wylie P. Clinical aspects of postirradiation sarcomas. *J Natl Cancer Inst* 1988; 80(4):233–40.

6 Laskin WB, Silverman TA, Enzinger FM. Postradiation soft tissue sarcomas. An analysis of 53 cases. *Cancer* 1988; 62(11):2330–40.

7 Tucker MA, D'Angio GJ, Boice JD Jr, *et al.* Bone sarcomas linked to radiotherapy and chemotherapy in children. *N Engl J Med* 1987; 317(10):588–93.

8 Swerdlow AJ, Douglas AJ, Hudson GV, *et al.* Risk of second primary cancers after Hodgkin's disease by type of treatment: analysis of 2846 patients in the British National Lymphoma Investigation. *Br Med J* 1992; 304:1137–43.

9 Levi F, Randimbison L, Te VC, La Vecchia C. Second primary cancers in patients with lung carcinoma. *Cancer* 1999; 86(1):186–90.

10 Bhatia S, Estrada-Batres L, Maryon T, *et al.* Second primary tumors in patients with cutaneous malignant melanoma. *Cancer* 1999; 86(10):2014–20.

11 Malmer B, Tavelin B, Henriksson R, Gronberg H. Primary brain tumours as second primary: a novel association between meningioma and colorectal cancer. *Int J Cancer* 2000; 85(1):78–81.

12 Eng C, Li FP, Abramson DH, *et al.* Mortality from second tumors among long-term survivors of retinoblastoma. *J Natl Cancer Inst* 1993; 85(14):1121–8.

13 Malkin D, Jolly KW, Barbier N, *et al.* Germline mutations of the p53 tumor-suppressor gene in children and young adults with second malignant neoplasms. *N Engl J Med* 1992; 326(20):1309–15.

14 Robinson E, Bar-Deroma R, Rennert G, Neugut AI. A comparison of the clinical characteristics of second primary and single primary sarcoma: a population based study. *J Surg Oncol* 1992; 50(4):263–6.

15 Dunkel IJ, Gerald WL, Rosenfield NS, *et al.* Outcome of patients with a history of bilateral retinoblastoma treated for a second malignancy: the Memorial Sloan–Kettering experience. *Med Pediatr Oncol* 1998;; 30(1):59–62.

16 Iscovich J, Boffetta P, Winkelmann R, Brennan P. Classic Kaposi's sarcoma as a second primary neoplasm. *Int J Cancer* 1999; 80(2):178–82.

17 Jacobsen GK, Mellemgaard A, Engelholm SA, Moller H. Increased incidence of sarcoma in patients treated for testicular seminoma. *Eur J Cancer* 1993; 29A(5):664–8.
